# Utilization of biological variation data in the interpretation of laboratory test results – survey about clinicians’ opinion and knowledge

**DOI:** 10.11613/BM.2021.010705

**Published:** 2020-12-15

**Authors:** Humeyra Ozturk Emre, Fatma Hande Karpuzoglu, Cihan Coskun, Ebru Demirel Sezer, Ozlem Goruroglu Ozturk, Fatma Ucar, Hikmet Can Cubukcu, Fatma Demet Arslan, Levent Deniz, Mehmet Senes, Mustafa Serteser, Cevat Yazici, Dogan Yucel, Abdurrahman Coskun

**Affiliations:** 1 Department of Medical Biochemistry, Kahramanmaras Necip Fazil City Hospital, Kahramanmaras, Turkey; 2 Department of Medical Biochemistry, Acibadem Labmed Clinical Laboratories, Istanbul, Turkey; 3 Department of Medical Biochemistry, Haydarpasa Training and Research Hospital, Istanbul, Turkey; 4 Department of Medical Biochemistry and Metabolism Laboratory, Faculty of Medicine, Ege University, Izmir, Turkey; 5 Department of Biochemistry, Faculty of Medicine, Çukurova University, Adana, Turkey; 6 Department of Clinical Biochemistry, Diskapi Yildirim Beyazit Training and Research Hospital, Ankara, Turkey^.^; 7 Department of Medical Biochemistry, Maresal Cakmak State Hospital, Erzurum, Turkey; 8 Department of Medical Biochemistry, University of Health Sciences, Tepecik Training and Research Hospital, Izmir, Turkey; 9 Department of Medical Biochemistry, University of Health Sciences, Istanbul Training and Research Hospital, Istanbul, Turkey; 10 Department of Medical Biochemistry, University of Health Sciences, Ankara Training and Research Hospital, Ankara, Turkey; 11 Department of Medical Biochemistry, School of Medicine, Acıbadem Mehmet Ali Aydınlar University, Istanbul, Turkey; 12 Department of Medical Biochemistry, Faculty of Medicine, Erciyes University, Kayseri, Turkey

**Keywords:** biological variation, laboratory error, patient safety, reference change value

## Abstract

**Introduction:**

To interpret test results correctly, understanding of the variations that affect test results is essential. The aim of this study is: 1) to evaluate the clinicians’ knowledge and opinion concerning biological variation (BV), and 2) to investigate if clinicians use BV in the interpretation of test results.

**Materials and methods:**

This study uses a questionnaire comprising open-ended and close-ended questions. Questions were selected from the real-life numerical examples of interpretation of test results, the knowledge about main sources of variations in laboratories and the opinion of clinicians on BV. A total of 399 clinicians were interviewed, and the answers were evaluated using a scoring system ranked from A (clinician has the highest level of knowledge and the ability of using BV data) to D (clinician has no knowledge about variations in laboratory). The results were presented as number (N) and percentage (%).

**Results:**

Altogether, 60.4% of clinicians have knowledge of pre-analytical and analytical variations; but only 3.5% of them have knowledge related to BV. The number of clinicians using BV data or reference change value (RCV) to interpret measurements results was zero, while 79.4% of clinicians accepted that the difference between two measurements results located within the reference interval may be significant.

**Conclusions:**

Clinicians do not use BV data or tools derived from BV such as RCV to interpret test results. It is recommended that BV should be included in the medical school curriculum, and clinicians should be encouraged to use BV data for safe and valid interpretation of test results.

## Introduction

Clinicians make many decisions based on laboratory tests results. Therefore, safe and valid interpretations of test results are essential for the correct diagnosis, monitoring, and treatment of patients. However, it has been shown that the rate of errors related to the interpretation of test results is high and ranked second among the five phases of the total testing process (TTP) (*1*-*3*). To interpret test results correctly, it is imperative for clinicians to understand the factors that affect these results. Laboratory test results are not fixed numbers; they vary mainly because of two sources of variations: laboratory-related (pre-analytical and analytical variations (CVa)) and biological variations (BVs). Laboratories can use standard procedures to minimize pre-analytical and CVa; nevertheless, BVs are specific to measurands and are not related to the procedures or instruments used in the laboratories. Therefore, they should be considered in the interpretation of the test results.

Reliable data are essential in the application of the BV of measurands in both laboratory and clinical practices. The European Biological Variation Study (EuBIVAS) has been designed by the European Federation of Clinical Chemistry and Laboratory Medicine (EFLM) Working Group on Biological Variation to deliver reliable BV estimates of measurands (*4*, *5*). Additionally, the EFLM Task Group on the BV database has developed the BV Data Critical Appraisal Checklist (BIVAC) to assess the quality of BV publications (*6*). They have also launched the EFLM Biological Variation Database (*7*). The database is updated when a new publication related to the BV of measurands is available.

Biological variation has two main components: within subject BV (CV_I_) defined as the fluctuation of a measurand around its homeostatic set point, and between subjects BV (CV_G_) defined as the variation between the homeostatic set points of different healthy subjects (*8*). Biological variations data have been widely used to: (i) set the analytical performance specifications (APS) such as those for CVa and bias of tests; (ii) calculate the index of individuality (II) for the evaluation of the utility of population-based reference intervals (RI); (iii) calculate the reference change value (RCV) for the evaluation of the significance of changes between consecutive measurements of different samples obtained from the same subject (*8*).

Although the concept of BV with its practical applications was developed mainly by laboratory specialists, its use is not limited to the laboratory medicine. Moreover, although the APS derived from the BV data is applied in the laboratory to ensure that the measurement methods fit the purpose, II and RCV are of much importance to clinicians than the laboratory. Additionally, various critical concepts such as RIs, delta checks, measurement uncertainty *etc.* were developed in laboratories. All these parameters should be considered in interpretation of test results. Since most of these concepts are based on ‘biological variation’, the knowledge and opinion of clinicians about biological variation can be used to analyse the possible root cause of misinterpretation of laboratory test results by clinicians. Therefore, BV may be a good model to examine how the information produced by the laboratory professionals is used by clinicians and the quality of communication between the laboratory specialists and the clinicians. The aim of this study is: 1) to evaluate the clinicians’ knowledge and opinion concerning BV, and 2) to investigate if clinicians use BV in the interpretation of test results.

## Materials and methods

This study is conducted by the Turkish Biochemical Society Biological Variation Working Group to evaluate the knowledge and experience of clinicians regarding the BV in six different regions in Turkey (Ankara, Istanbul, Adana, Izmir, Kayseri, and Nigde). The survey took place from June till December 2018. A questionnaire that is theoretically and practically related to BV was prepared.

### Questionnaire design and administration

The questionnaire comprises two types of questions. Five questions were open-ended (Questions 1-5), and they were prepared to assess whether clinicians use the BV concept and data when interpreting test results (Table 1). Three questions were close-ended (Questions 6-8), and they were prepared to evaluate clinicians’ awareness of BV (Table 2).

**Table 1:** Open-ended questions used to assess whether clinicians use biological variation (BV) concept and data when interpreting test results

**Rationale**	**Question**	**Score designation**	**Answer**
To assess how clinicians evaluate consecutive measurements when one of the results of a test is within the RI and the other is out of RI.	1. The pre-treatment alanine transaminase (ALT) value of a female patient whom you prescribed medication with possible side effects on the liver was 40 IU/L, while her post-treatment ALT value was found to be 60 IU/L. (ALT RI: 7–45 IU/L) How would you figure out a significant difference between these two measurements?	A	S/he performs a mathematical calculation (includes Delta Check and RCV and makes calculations) to figure out whether there is any significant difference between the two measurements.
		B	S/he believes that the difference between the two measurements may originate from the biological and analytical/pre-analytical variations; however s/he fails to calculate the Delta Check and RCV.
		C	S/he is aware of the pre-analytical and/or analytical variation. (Results from the same instrument may vary).
		D	S/he considers that the difference between the consecutive measurements originates from laboratory errors.
To assess how clinicians evaluate consecutive measurements while the results of both measurements of a test are within the RI.	2. The total cholesterol result of one of your patients was 140 mg/dl while it was found to be 190 mg/dl in the subsequent quarterly check (RI < 200 mg/dl). What is your assessment on the 50 mg/dl difference between these two results that are within the RI?	A	S/he makes assessment by comparing the Delta Check and the RCV values.
		B	S/he believes that there may be significant variations among the results because of the random biological or analytical/pre-analytical variations even when both of them are within the RI.
		C	S/he finds the variation between the test results as significant. However, s/he believes that this is likely to result from prescribed drugs and/or work/lifestyle.
		D	S/he accepts any variation of test results within the reference range as normal.
To measure the knowledge of clinicians on the variables that affect test results.	3. Which factors do you think may influence the variations observed between the measured results of the test?	A	The lab results of a test may vary. Such variations may result from pre-analytical, analytical and/or random biological variation.
		B	S/he considers the clinical, pre- analytical or analytical variations.
		C	S/he considers only the laboratory-originated variations.
		D	Unless the patient’s clinic is changed, there should not be any variations in the test results, or has no idea.
To measure the knowledge of clinicians on the biological variations of tests.	4. What do you think about the biological variations of the laboratory tests?	A	Lab test results naturally vary on a range. Such variations occur intra-individually and inter-individually.
		B	S/he knows such variations are common in test results. However, s/he does not know the intra-individual and inter-individual random variations.
		C	S/he knows biological variations such as age, gender, ethnicity, menstrual cycle, seasonal variations, and diurnal variation may affect the test results; however, s/he does not have information on the random biological variations.
		D	S/he does not think that the variations between test results may arise from biological factors. i. S/he does not take the test results into consideration as long as they are within the RI. ii. S/he believes that the variations in the test results result from pre-analytical reasons (hunger, fullness, haemolysis, *etc.*) iii. No idea.
To measure whether clinicians consider the biological variations of tests when interpreting their results.	5. In general, do you take biological variation into account in the interpretation of laboratory results?	A	S/he considers biological variation when s/he assesses the difference between test results in the case of consecutive measurements. S/he is able to perform numeric calculations.
		B	S/he is aware that random biological factors have a role in the test result’s variations. S/he is unable to make the calculations.
		C	S/he is aware that random biological factors affect the test results’ variations. However, s/he believes that they result from biological variables such as age, gender, menstrual cycle, *etc*.
		D	S/he thinks it is not necessary to take biological variation into account when interpreting lab results.
RI - reference intervals. RCV - reference change value.			

**Table 2:** Close-ended questions used to assess whether clinicians use biological variation (BV) concept and data when interpreting test results

**Rationale**	**Question**	**Answer**
To assess whether clinicians read publications on biological variation.	6. Have you read any publication on intra-individual and inter-individual biological variations of measurands?	a) Yes b) No
To assess whether clinicians follow the scientific activities on biological variation.	7. Have you ever taken any course, or had training on intra-individual and inter-individual biological variation of the measurands?	a) Yes b) No
To assess whether clinicians consider the concept of biological variation necessary for the future generations of clinicians.	8. Do you think biological variation should be included in the medical curriculum?	a) Yes b) No

A total of 400 clinicians were invited and 399 accepted to participate in the survey. Face-to-face interviews of all the clinicians were conducted by the laboratory specialists. Instead of sending questionnaires to clinicians, we preferred meeting them in person to increase the reliability of the results and to prevent any potential variation. Providing any clue to clinicians for the correct answer of the questions, or any information on the definition of BV was finically avoided until the end of the questionnaire. The clinicians were divided into four main categories: paediatricians, internal medicine specialists, surgical medicine specialists and general practitioners.

### Evaluation of the answers given by clinicians

Evaluating the answers of the open-ended questions is not as easy as the multiple choice questions. Therefore, for each open-ended question, a scoring system that ranged from A to D (Table 1) was used. In general, A indicated the highest level of knowledge and the ability to effectively use the BV data, B indicated that the clinician has theoretical knowledge about BV; however, he/she lacks the ability to use it in practice, C indicated that the clinician has a fair idea of variation in laboratories but not particularly BV, and D indicated that clinician has no knowledge of any variation in laboratory. The detailed explanations of the indicators are given in Table 1. Every open-ended question was independently evaluated by two medical biochemists to avoid bias. If the evaluations did not match, a third medical biochemist was involved, and the final decision was taken.

### Statistical analysis

Chi-squared test was used to evaluate the significance among groups. Values of P < 0.05 were accepted as statistically significant. Statistical analysis was performed using MedCalc statistical software (MedCalc Software Ltd, Ostend, Belgium).

## Results

The demographic characteristics of the participants are shown in Table 3. Altogether, 399 clinicians were interviewed. More than 98% of clinicians had more than a year’s experience, and 30% had more than 10 years of experience. Most of the clinicians (91%) participating in the study were academically active (*i.e*., they were affiliated to universities and research and training hospitals).

**Table 3:** Demographic characteristics of clinicians who participated in the study

**Characteristics**	**Variable**	**N (%)**
Sex	Male	164 (41.1)
	Female	235 (58.9)
Age (years)	< 25	7 (1.8)
	25–29	190 (47.6)
	30–39	104 (26.1)
	40–49	69 (17.3)
	50–59	24 (6.0)
	> 59	5 (1.2)
Experience	< 1	7 (1.8)
(years)	1-3	118 (29.5)
	3-5	108 (27.1)
	5-10	45 (11.3)
	> 10	121 (30.3)
Hospital	University (Public)	158 (39.6)
	University (Foundation)	25 (6.3)
	Research and Training Hospital	181 (45.4)
	Public Hospital	8 (2.0)
	Private Hospital	26 (6.5)
	Other	1 (0.2)
Category	Paediatricians	120 (30)
	Internal Medicine Specialists	206 (51)
	Surgical Medicine Specialists	28 (7)
	General Practitioners	45 (12)
Total		**399 (100)**

### Use of biological variation data by clinicians in interpreting test results

**Figure 1:**
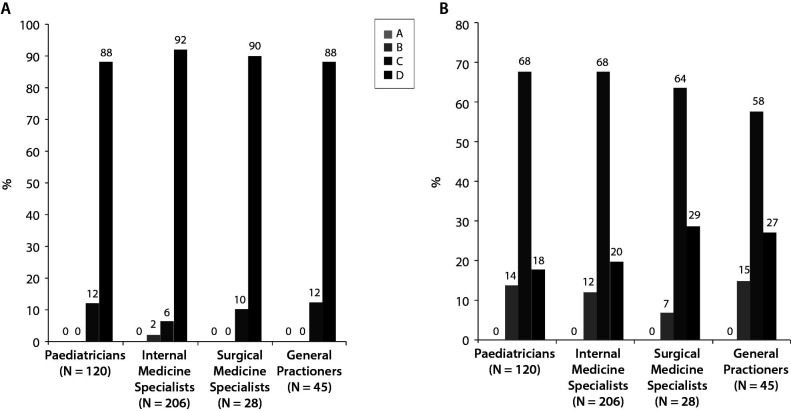
Scores of clinicians evaluating consecutive measurements. A) One of the results of a test being within the reference interval and the other being out of reference interval (Q1) (P = 0.285); B) Results of both measurements of a test being within the reference interval (Q2) (P = 0.677). The indications of A, B, C and D are given in Table 1.

As shown in Figure 1 A, neither of clinicians used RCV to evaluate the difference between consecutive measurements when one of the results of a test is within the RI and the other is out of the RI (Table 1, Question 1 (Q1)). Furthermore, except three internal medicine specialists (0.75% of all study participants), the clinicians had not detailed information about BV concept.

Similarly, neither of clinicians used RCV to evaluate the difference between consecutive measurements while the results of both measurements of a test are within the RI (Table 1, Q2). However, 79.4% of clinicians accepted that even if the results of the two consecutive measurements are located within the RI, the difference between these two measurements may be significant. The reason for the difference was considered to be related to diet or the prescribed drugs by 66.7% clinicians and to laboratory-related variations by 12.8% clinicians. Moreover, 20.6% of the clinicians did not consider this difference as significant (Figure 1 B).

**Figure 2:**
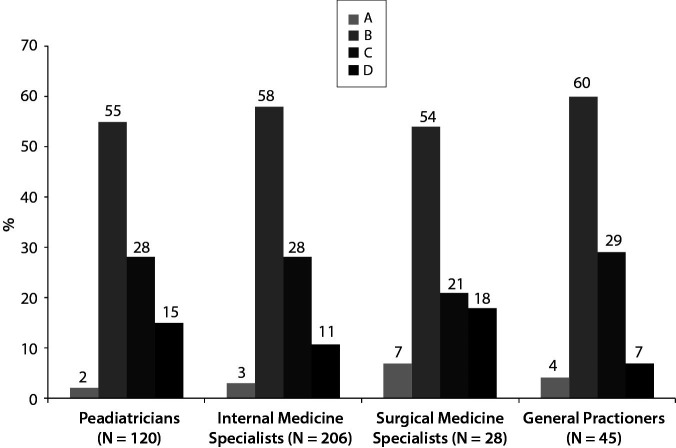
Scores of clinicians’ knowledge about variables that affect test results (Q3) (P = 0.838) . The indications of A, B, C and D are given in Table 1.

Although clinicians mainly do not use RCV to evaluate the difference between consecutive measurements, they have knowledge that laboratory test results are not fixed numbers; and some variables affect test results (Table 1, Q3). A total of 60.4% of the clinicians had knowledge of pre-analytical and analytical variations; however, only 3.5% of them had awareness related to BV (Figure 2).

**Figure 3:**
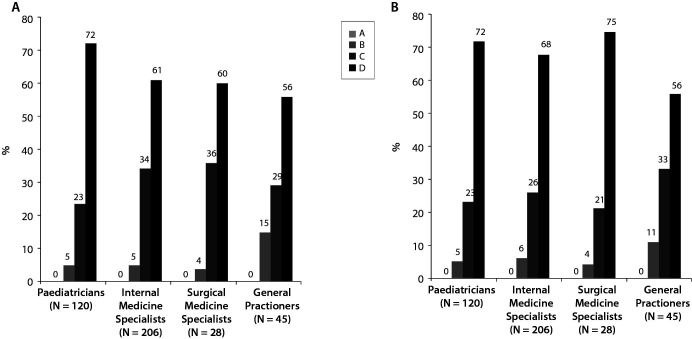
Scores of clinicians’ A) knowledge about biological variations of tests (Q4) (P = 0.211) B) using the biological variations of tests when interpreting their results (Q5) (P = 0.459) . The indications of A, B, C and D are given in Table 1.

In question 4, we examined the opinions of clinicians on BV, and question 5 was intended to determine the rate at which clinicians take BV into consideration when evaluating test results. Question 5 was evaluated in connection with the Question 4. In Question 4, 60.9% of clinicians were scored as D and 0% of clinicians were scored as A (Figure 3 A) *i.e*. neither of clinicians were familiar with the within- and between-subject biological variation. The similar trend was observed in Question 5 (Figure 3 B).

### Clinicians’ awareness of biological variation

**Figure 4:**
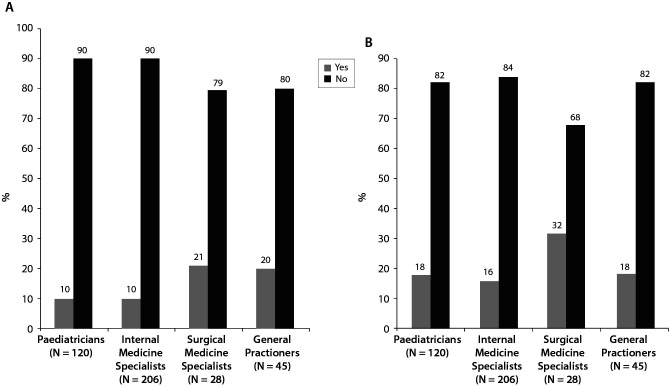
Scores of clinicians whether they A) read publications on biological variation (Q6) (P = 0.066) B) follow scientific activities on biological variation (Q7) (P = 0.226).

**Figure 5:**
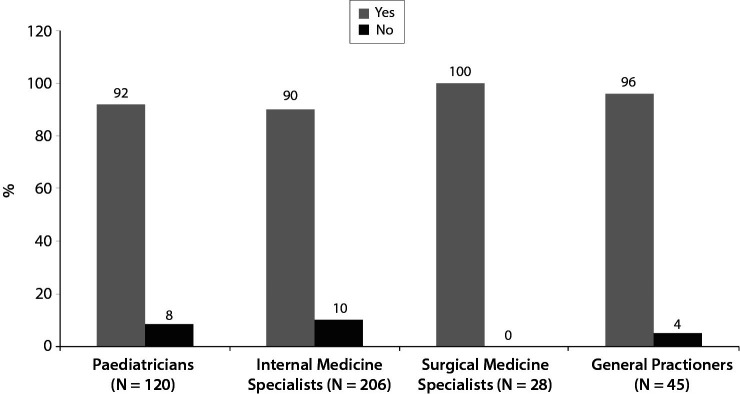
Scores of clinicians considering the concept of biological variation necessary for the future generations of clinicians (Q8) (P = 0.204).

Firstly, 88.5% of the clinicians stated that they had not read any article on BVs (Q6) (Figure 4 A). Further, 82.0% of the clinicians reported that they did not recall anything on this subject from their medical training (Q7) (Figure 4B). Considering the last question, 92.0% of the clinicians stated that BV had to be covered by medical education and training for the future generations of clinicians (Figure 5).

## Discussion

In this study, the knowledge, experience and clinicians’ awareness about BVs of measurands have been evaluated. Although within the last decade BV has attracted the attention of researchers in laboratory medicine, it also has the great potential to be used by clinicians to the benefit of patients.

Medical errors are one of the serious causes of morbidity and mortality (*9*, *10*). Recently, Makary *et al.* reported that after cardiopulmonary and malignant diseases, medical errors were the third leading cause of death in the US (*11*). Most laboratory-related medical errors originate from physicians who order and interpret test results (*i.e*., the initial and final steps of TTP) (*1*-*3*, *12*, *13*). To achieve efficiency and quality in the laboratory processes, errors should be identified and subsequently reduced to a negligible level. We think that using BV by clinicians in interpretation of test results will decrease medical errors, particularly post-post analytical errors.

It is observed that the presence of one (Q1) or two (Q2) consecutive measurement results within the RI did not change the clinicians’ interpretation methods of the test results. In other words, clinicians do not use RCV to evaluate the difference between consecutive measurements. According to the majority of clinicians, the reason for the difference between two measurements results depends on the patients’ life style, the side effects of prescribed drugs or laboratory-related errors. In this study, it is shown that if there are no clinical findings, clinicians usually do not take into account the changes that slightly exceed the RIs, as given in the alanine transaminase (ALT) example (Q1). ACG clinical guideline recommends that ‘in case of clinical findings, even in the absence of abnormal liver chemistries an evaluation should be initiated’ and < 2x upper reference limits of ALT elevation is accepted as borderline (*14*). As given in the cholesterol example (Q2), despite a distinct difference, even if the two measurement results are within the RI, the difference is not usually taken into account by clinicians. Such differences are often attributed to patients’ lifestyle or other factors.

Monitoring of patients’ test results plays a crucial role in the evaluation of prognosis, the effectiveness of ongoing treatment and detection of possible recurrences. The II of most of measurands are lower than 1, which means that conventional RI is less effective in monitoring of patients results (*7*). Reference change value plays a central role in objective monitoring of test results, and in daily practice it should be used by clinicians as frequently as RI. Using ‘flags’ on reports of laboratory results, indicating significant changes between consecutive measurements, might help clinicians to interpret laboratory results correctly.

Contrary to BV, clinicians are partly familiar with laboratory-related variations such as pre-analytical and analytical variations (Q3 and Q4) probably because of the undergraduate and/or postgraduate clinical biochemistry education. This shows the importance of the content of the curriculum. Smith *et al.* recommended a curriculum including how to use the RIs to interpret test results, and a detailed knowledge on variations that affect test results such as variability in repeated measurements as well as variability within and between individuals (*15*). Thue and Sandberg showed that the primary care physicians are not familiar with BV and its usage in clinical practice (*16*). Similarly, this study showed that clinicians were not familiar with the concept of BV (Q4) and its clinical application (Q5). However, this was the case for both the primary care physicians and all the different specialties.

In general, the clinicians’ answers showed that they do not follow the literature in the field of BV. This may be explained by the limited number of articles published in clinical journals. Laboratory societies should take initiatives to include BV and its related topics in medical education and training. A research conducted in the US in 2014 indicated that laboratory medicine education was not sufficient in medical education, and it was necessary for proper test ordering and interpretation by physicians (*17*). In our study, clinicians reported that they had not received any training on BV; nevertheless, they thought that it should be part of medical education. Laboratory specialists have to be instructors and consultants (*18*, *19*). They should provide guidance to clinicians on laboratory-related information. One of the methods may be the use of BV data in routine clinical practice.

Improvement in collaborations between laboratory specialists and clinicians may result in a widespread clinical use of BV. Clinicians could then identified the clinical situations where BV data should be used for the benefits of patients.

In conclusion, clinicians do not use BV data and RCV to interpret tests results. It should be noted that although RCV was investigated and developed by laboratory specialists, its usage concerns clinicians rather than laboratory specialists. Effective communication/collaboration between laboratory specialists and clinicians will enable clinicians to interpret laboratory tests correctly, and use BV data more efficiently. Laboratory specialists have a crucial role in communicating with clinicians on BV. Some of the ways to achieve this include:

1. Clinicians should be encouraged to use RCV to interpret consecutive measurements results.
2. Education and training programs should be provided to clinicians to improve their knowledge and experience.
3. Biological variation and its usage should be added to the medical school curriculum.
4. Laboratory specialists should become more focused on taking an active role as consultants.

In this knowledge-intensive era, guiding clinicians to interpret test results correctly should become one of our priorities along with managing the performance of laboratories and providing high-quality and accurate results.

This study was conducted in only one geographic region and this is the main limitation of the study. Multinational studies are necessary to make valid conclusions.
